# HIV-Stigma in Nigeria: Review of Research Studies, Policies, and Programmes

**DOI:** 10.1155/2017/5812650

**Published:** 2017-12-27

**Authors:** Clifford O. Odimegwu, Joshua O. Akinyemi, Olatunji O. Alabi

**Affiliations:** ^1^Demography and Population Studies Programme, Schools of Public Health and Social Sciences, University of the Witwatersrand, Johannesburg, South Africa; ^2^Department of Epidemiology and Medical Statistics, Faculty of Public Health, College of Medicine, University of Ibadan, Ibadan, Nigeria; ^3^Department of Demography and Social Statistics, Federal University, Birnin Kebbi, Nigeria

## Abstract

Nigeria has about 3.8 million people living with HIV, the second largest globally. Stigma and discrimination are major barriers to testing, treatment uptake, and adherence. In this review, we synthesized information on research studies, policies, and programmes related to HIV-stigma in Nigeria. This was with a view to identify critical areas that research and programmes must address in order to accelerate the progress towards zero (new infections, discrimination, and death) target by year 2030. Existing studies were mostly devoted to stigma assessment using varieties of measures. Research, policies, and programmes in the past two decades have made very useful contributions to stigma reduction. We identified the need for a consistent, valid, and objective measure of stigma at different levels of the HIV response. Nigeria does not lack relevant policies; what needs to be strengthened are design, planning, implementation, monitoring, and evaluation of context-specific stigma reduction programmes.

## 1. Introduction

One of the successes recorded in the Millennium Development Goals was target 6A which aimed to halt HIV by 2015 and begin to reverse its spread. As of 2014, the number of new HIV infections and AIDS-related deaths has declined by 40% and 42%, respectively [1]. Sub-Saharan Africa region was not left behind in this achievement despite the fact that the region is home to 70.0% of persons living with HIV globally [1]. This progress has been attributed to massive improvement in funding which enhanced wider coverage of antiretroviral treatment, innovative approaches to treatment, and prevention interventions [1]. Some challenges still remain which include the need to attain universal treatment coverage and eliminate new infections; treatment of comorbidities; stigma; and discrimination [2]. To confront these challenges and build on the laudable progress so far recorded, one of the health targets under the new Sustainable Development Goals (SDG) is to end the AIDS epidemic by 2030. In pursuit of this, the Joint United Nations Program on HIV/AIDS (UNAIDS) has proposed a global strategy whose target is reducing the number of new HIV infections by 90% and number of AIDS-related deaths by 80% of the level at year 2010 [1]. In fact, UNAIDS has a new vision of “zero new HIV infections, zero discrimination, and zero AIDS-related deaths” [3].

Meanwhile, recent statistics shows that 36.7 million persons were living with HIV as of the end of 2015 of which about 46% have access to treatment [3]. Of these, Nigeria has the second highest number, 3.8 million with adult prevalence of 3.1% [4]. About 44% of adults and children living with HIV have access to ART based on the eligibility criteria of CD4 count of 350 cells/mm^3^. With the largest population in SSA, only 26% of its people have ever done HIV testing [4]. HIV epidemiology in Nigeria indicates that infections are higher among women while prevalence varied across the six geopolitical regions with the highest rate in the South South (5.5%). One of the main factors driving the infection in Nigeria includes low risk perception, concurrent sexual partnerships, and inadequate access to quality healthcare services [4]. At the centre of these factors is the challenge of HIV-stigma and discrimination which are a major barrier militating against the national response to the epidemic.

According to UNAIDS 2015 guidelines on terminologies, stigma refers to beliefs and/or attitudes marking or staining a person or group of people as unworthy or discreditable [5]. Discrimination results from stigma when any form of distinction, exclusion, or restriction is displayed against an individual because of an attribute or a personal characteristic. For operational and programmatic purposes, HIV-related stigma is described as “negative beliefs, feelings, and attitudes towards people living with HIV (PLHIV), groups associated with PLHIV and other key populations at higher risk of HIV infection” [2]. HIV-related discrimination is the “unfair and unjust treatment (act or omission) of an individual based on his or her real or perceived HIV status.” Stigma Stigma and discrimination are formidable threats to the success of HIV care and treatment programs. Several studies have shown stigma and discrimination to affect HIV testing [6], disclosure of serostatus [7], retention, and adherence to treatment [8]. Meta-analysis of 64 studies conducted in different settings demonstrated significant effects of HIV-stigma on mental health, quality of life, use of health services, and physical health of PLHIV [9]. Manifestations or expression of stigma is influenced by sociocultural, political, and economic factors all of which translates into different forms of inequalities in access to HIV care, treatment, and support [10]. The consequence of these multilevel influences is that HIV-stigma operates at individual, family, community, and institutional levels and many of these factors also varied across cultures, thereby necessitating context-specific strategies to address the phenomena.

In congruence with the advancement in the global response, research and programmes on HIV-related stigma have witnessed improvement since the entrance of HIV into public health discourse. For instance, beyond the initial complexities associated with definition, measurement, impact assessment, and reduction of HIV-stigma [11], empirical measures for different domains of stigma have been proposed and validated in some sub-Saharan African countries [12]. Systematic reviews and other forms of research studies have provided evidence on various interventions to reduce HIV-stigma [13–15]. Examples of these interventions which have dominated the HIV-stigma reduction efforts include information-based approaches such as behaviour change communications; capacity building (training of healthcare providers and other allied workers); counselling and support for PLHIV (support groups and network); and involvement of PLHIV in different activities as a way of humanizing the infection such that it is seen as any other chronic health condition.

Nigeria as one of the countries with the largest number of PLHIV has strived to combat HIV-stigma and discrimination to curtail the epidemic. The early (1990s) and peak years (mid-2000s) of the epidemic in Nigeria were characterised by various forms of stigma and discrimination at different levels of human interaction [16]. A review of studies on HIV-related stigma in Nigeria between 1987 and 2008 found eight peer-reviewed articles [17]. The main findings include the following: HIV-stigma manifests mostly as negative attitudes against PLHIV by individuals and community members and unwillingness to treat PLHIV by healthcare workers. The potential of educational interventions to reduce HIV-related stigma was also demonstrated among secondary school pupils, students in nursing schools, and practicing healthcare workers. Some critical gaps identified by the review were absence of coherent and consistent measure of stigma across studies and near absence of intervention studies on stigma reduction [17]. An obvious gap in the previous review of HIV-stigma in Nigeria was the lack of evidence on the experiences of stigma among PLHIV. Furthermore, almost one decade after the review, the national response to HIV in Nigeria has experienced positive shifts in diverse ramifications. For instance, information and awareness about HIV are almost universal; political will and stakeholder involvement have become more robust [4]. It is also expected that research work on HIV-stigma must have advanced beyond the level it was in 2007. Therefore, this paper is aimed at reviewing studies, policies, and programmes related to HIV-stigma in Nigeria. We synthesized data and information on these issues and identified critical areas that research and programmes must address to accelerate progress towards zero (new infections, discrimination, and death) target set by UNAIDS.

## 2. Methods

In March 2017, we searched electronic databases for articles on HIV-stigma in Nigeria between 1999 and 2016. The databases were PubMed, African Journal Online, and JSTOR. Search terms involved different combination of the following: HIV, AIDS, Stigma, Discrimination, and Nigeria. The word “attitude” was also used as a search term because many of the related articles used it to connote stigma or discrimination. Abstracts of each article in the search results were reviewed to determine whether they met criteria for inclusion in the review. For an article to be included in the review, it must address HIV/AIDS stigma or discrimination as a dependent or independent variable. Inclusion was not restricted to any study design; therefore, both quantitative and qualitative studies that addressed HIV-stigma and/or discrimination in Nigeria were selected. Full text of articles that met the inclusion criteria were retrieved and reviewed. For each reviewed article, information was extracted on authorship and year of publication; objective; research design; how stigma was measured (and whether as a dependent or independent variable); and the main findings. These details were summarised in a Table 1. Since this review also includes policies and programmes related to HIV-stigma in Nigeria, we searched the website of relevant organisations for documents related to the subject of interest. Such organisations included the National AIDS Control Agency (NACA), Federal Ministry of Health and Joint United Nations Programme on HIV/AIDS (UNAIDS), Network of People Living with HIV in Nigeria (NEPWHAN). References of retrieved articles, policy documents, and programme reports were also physically scrutinized.

## 3. Results

**Figure 1:**
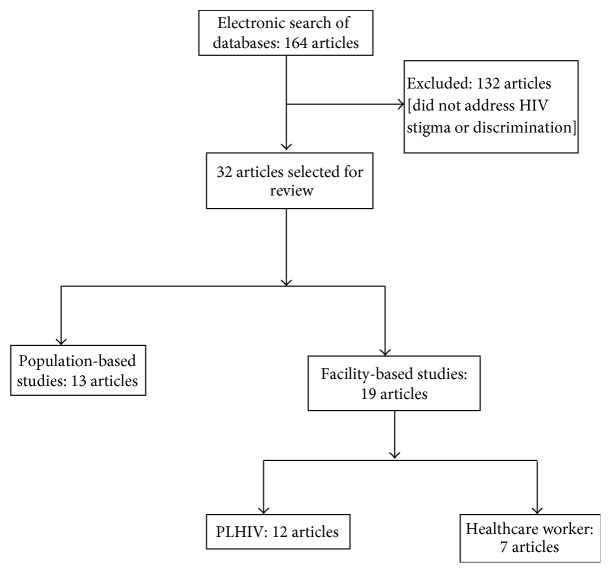
Flow chart of articles selected for review.

Compared to the previous review by Monjok et al. [17], we found greater number of research articles related to HIV-stigma in Nigeria.  Figure 1 shows the search results, exclusions, and the number of articles eventually included in the review. Of the 32 articles reviewed, 13 were population-based studies. The remaining 19 articles were based in health facilities (11 among PLHIV; 1 among men who have sex with men [MSM]; and 7 among health workers). Apart from 6 articles with national representation, majority of the others (especially those that utilised qualitative techniques) were based in the Southern regions of Nigeria. About 60% of all articles used quantitative techniques while qualitative papers constituted 21.9%. Substantive findings from review of research articles are summarised according to the study population: general population (community members); healthcare workers; and people living with HIV (PLHIV).

### 3.1. Population-Based Studies of HIV-Stigma

Many population-based studies in Nigeria have attempted to measure HIV-related stigma using attitudinal questions as proxy. A tenuous challenge that emanates from many of these studies was that the attitudinal questions used to represent stigma were rarely the same across different studies. One of the earliest community-based research on HIV-stigma in Nigeria was a mixed-method study conducted in South West and South East Nigeria in the early 2000s [18] but published in 2013 [19]. Seven dimensions of HIV-stigma were measured using questions about attitudes of community members towards anyone living with HIV. These dimensions were negative feelings, coercive attitudes, attribution of blame, avoidant behaviours, symbolic contact, interaction with PLHIV, and attitude to an infected partner. Results showed that there was a high prevalence of stigma and discrimination across these domains and stigma was also found to be associated with low uptake of voluntary counselling and testing. It was as high as 85% for avoidant behaviours. Participants from the South Eastern region exhibited stigma more than those from the South West [19]. Negative relationship between HIV-stigma and VCT was also documented among youths in North Eastern and North Western part of Nigeria [20]. Adopting the collectivity of behaviour model, the authors investigated three dimensions of perceived stigma (labelling, relationship, and status disclosure) at individual and community levels. High level of stigma was associated with lower odds of VCT and the effect was even stronger at the community level. There were also gender differences with females showing greater level of stigma. Other population-based studies of HIV-stigma in Nigeria have used accepting attitude as indicator [21, 22]. Incidentally, these were based on nationally representative samples of men and women aged 15–59 and 15–49 years, respectively, with higher prevalence of stigma among women. Analysis of the 2005 National HIV/AIDS and Reproductive Health Survey (NARHS) data revealed that exposure to media information about HIV/AIDS was associated with increased knowledge about the infection and ultimately translated to better accepting attitude [22]. Regional differences in HIV-stigma were also observed despite adjustment for individual characteristics and community media exposure. Adebayo et al. [21] analysed data from the NARHS 2003–2007 to provide evidence on improvement in the level of accepting attitude in Nigeria over this period. In a recent analysis of Nigeria Demographic and Health Survey (NDHS) 2013 data, Dahlui et al. [23] showed that about half of Nigerians still exhibited stigmatizing attitude against PLHIV. This was found associated with younger age, urban residence, lack of formal education, and poor household wealth quintile. The measure of stigma was two attitudinal questions which focused on blaming and shaming of persons living with HIV.

Fertility desires among people living with HIV have attracted attention of researchers especially with improved quality of life offered by ART [24]. To fill important gap about the attitude of people to reproductive decisions of HIV-positive women, a community-based study was conducted in Nigeria and Zambia to describe attitudes towards abortion or childbirth by HIV-positive pregnant women [25]. The Nigeria arm of the study was conducted in selected enumeration areas in Lagos (South west), Enugu (South east), Benue (North Central), and Kaduna (North West). Findings revealed that the level of stigma against birth or abortion by HIV-positive pregnant women was low and similar between men and women. Lack of formal education was the main factor independently associated with stigmatizing attitude towards abortion or childbirth by HIV-positive woman [25]. Analysis was not stratified by state because of the multicountry nature of the study. Therefore, it is not known whether there were variations across the four states (regions) where the study was conducted in Nigeria.

Few of the population-based studies of HIV-stigma in Nigeria have some semblance of interventions to assess the effectiveness of health educational intervention on stigma reduction. All the three studies in this category utilised a quasi-experimental design and showed that stigma reduction can be achieved using educational interventions. Although the interventions were conducted among market women [16], secondary school students [26], and student nurses [27], the efficacy of this approach in the larger population has been demonstrated in a field experiment [28] and analysis of repeated cross-sectional data [29].

Very few community-based qualitative studies of HIV-stigma were found in the course of the review. Incidentally, these were mostly based in Southern parts of Nigeria. Evidence from the south west revealed that social constructions of HIV-stigma are deeply rooted in cultural beliefs and perceptions about the lifestyle of those infected [16, 19]. A cross-national textual analysis of narratives from a writing contest among youths was conducted to compare social representation of symbolic stigma in six SSA countries including Nigeria [30]. The findings pointed to the fact that social representation of stigmatizing attitudes often pertained to the circumstances around the process of HIV infection though there were variations across the six sociocultural contexts analysed. Negative views about PLHIV were more predominant in low HIV prevalence settings. The study further revealed that Nigerian participants who were selected from the South Eastern part displayed the highest cases of symbolic stigma [30]. To shed more light on the gender differences in HIV-stigma, Mbonu et al. [31] adopted Cornell's theory of gender and power to explore power-related differences between men and women in their reactions to PLHIV at family, community, and institutional level. In-depth interviews were conducted among community members, healthcare providers, and PLHIV. The study also showed that stigmatizing behaviour was worst against women and was a reflection of the subsisting power imbalance that exists in patriarchal societies such as Nigeria. This imbalance manifested in forms of financial inequality, authority, and structural norms.

### 3.2. Facility-Based Studies of HIV-Stigma in Nigeria

The second group of studies on HIV-stigma found to be common in Nigeria were those conducted in health facilities. As expected, the study population were either healthcare providers or PLHIV who receive care and treatment from the facilities. An important observation about many of these studies was that they were domiciled mostly in facilities providing specialist or tertiary care and as such were in urban areas. Findings from the review of these studies are summarised according to the study population, healthcare workers and PLHIV.

#### 3.2.1. Healthcare Workers

Majority of the studies among healthcare workers were conducted around early and mid-2000s. This was a time that could be referred to as the peak of the HIV epidemic in Nigeria. Virtually all of them were aimed at accessing attitudes towards care and treatment of PLHIV. The earliest among these studies was a quasi-experimental study in South South Nigeria by Ezedinachi et al. [32]. They documented unwillingness to treat, fear, and avoidance of PLHIV among clinicians. Following educational interventions, data collected one year later showed there was significant decline in stigmatization and discriminatory behaviour towards PLHIV [32]. Preponderance of stigma against PLHIV was very common in all the other studies found in this category and this was similar among clinicians [33], dental and oral health professional [34–36], nurses, and medical lab scientists [37]. In anticipation of the potential roles of primary healthcare workers in care and treatment of PLHIV, a study in North Central Nigeria showed similar findings of a high level of stigma which often manifest in form of fear of casual transmission, shaming and blaming, discrimination, and status disclosure [38].

### 3.3. Stigmatization Experiences among People Living with HIV

The second group of facility-based studies are those that documented stigmatization and discrimination experiences among PLHIV. Prevalence of stigmatizing experiences varied widely, ranging from 8% to 60%. This wide variation is due to the diverse indices/measures used for stigma. In fact, no two studies defined or measured HIV-stigma in the same manner. This posed limitations to study comparisons and generalisation from one study setting to another. As observed earlier, majority of the studies in this category were conducted at tertiary health facilities in Southern Nigeria except one at a Teaching Hospital in North Central region by Owolabi et al. [39]. In the paper, 25% of ART patients reported that they had experienced stigma or discrimination at family, hospital, and community levels and at the workplace. Forms of stigma experienced included blaming for being HIV-positive and name calling (prostitute, skeleton, and unfaithful person among others). Examples of discriminatory practices against PLHIV were hospital (selective use of gloves, poor quality care, and isolation from other patients); community (social isolation, restriction in family events, and hostility); family (not sharing of cutleries and toilet facilities, breach of confidentiality); workplace (threat of employment termination, isolation by coworkers). Adapting the Berger stigma scale [40], forms of stigma experienced by PLHIV were also documented in a cross-sectional study conducted at another tertiary facility in South East Nigeria [41]. These were personalised/internalised stigma, status disclosure, negative self-image, and concerns about public attitude. Females were found to experience higher degree of the first three dimensions. A weakness of the paper was that the stigma scale was treated as a continuous measure and scores were generated for analyses. It was therefore difficult to estimate the magnitude or prevalence of stigma in the four domains examined.

The burden of stigma among PLHIV may be higher than documented in quantitative studies as findings from FGD participants in Lagos, Nigeria, showed that almost 75% reported that life has become very traumatic due to stigmatization from friends, family members, healthcare workers, and workplace [42]. Studies have shown that stigma was associated with ART adherence and mental health status of PLHIV. For instance, low stigma level was found to be associated with good ART adherence among participants in a cross-sectional study at a tertiary facility in South West Nigeria [43]. Regarding the consequences of HIV-related stigma, Adewuya et al. [44] found that 27.3% of patients who had experienced stigmatizing events such as isolation and blaming had posttraumatic stress disorder (PTSD). The study was conducted at a private HIV/AIDS care facility in South West Nigeria. It is suspected that the situation could be worst in public health facility where there are larger patient population and therefore greater pressure on healthcare providers. Evidence from another study in South East region suggests that the prevalence of depression was 33.3% and this was associated with negative self-image domain on the Berger HIV scale [45]. Personalised or internalised stigma was also associated with lesser likelihood of status disclosure among PLHIV [46]. These limit the self-esteem and confidence among PLHIV with a negative consequence on their overall mental health status.

Quite a few qualitative studies have also explored the experiences of stigma and discrimination among PLHIV. Results from focus group discussion in Southern parts of Benue State in North Central region of Nigeria highlighted the high level of rejection and discrimination of PLHIV in the late 1990s [47]. Isolation and avoidance of those infected by HIV were a common practice fuelled by fear of being infected by “a disease without cure.” Community members believed that caring for anyone infected by HIV was waste of resources because HIV was more like a death sentence [47]. With better awareness and availability of ART, the negative perception has waned over time such that PLHIVs also desire to fulfil social expectations about marriage and reproduction [48]. Ethnographic case studies from South East Nigeria actually showed that marriage and reproduction by HIV-positive men and women are strategies used to confront the problem of HIV-stigma [48].

Drawing on Deacon's framework [49], a qualitative exploration of stigma before and after ART initiation revealed very interesting findings about the dynamic nature of stigma [50]. The central argument of the Deacon's framework was that stigma is a dynamic sociocultural phenomenon that changes in response to different situations [49]. Based on this premise, stigma could be classified as self-stigma, anticipated stigma, and enacted stigma. Before ART, there were experiences of self-isolation, fear of death, family withdrawal, rejection, and abandonment [50]. After ART commencement, with better or healthier physical appearance, family and community members began to accept PLHIV and status disclosure also improved. In sum, stigma was a motivator for treatment adherence which in turn reduced self-stigma, enacted stigma, and anticipated stigma [50]. This evidence needs to be further substantiated because participants in the study were recruited from a community-based organisation. If participants had been recruited from a public health facility, could the findings be different? Another exploratory study of stigma and survival challenges among ART patients at a secondary health facility in South West Nigeria revealed that stigmatizing experiences were low among family members but high from nonfamily members including health workers [51]. The study also showed that some well-intentioned social interventions (such as packaged food parcels for nutritional supplementation) for PLHIV ended up stigmatizing them. In the face of these daunting challenges of stigma and discrimination, a study in Lagos, South West Nigeria, documented the coping strategies that have been deployed by PLHIV. The most common were maintaining a low public profile by avoiding public functions, avoiding seeking care in public health facilities, and nondisclosure of serostatus [42]. Of course, these approaches do not augur well for universal coverage of care, treatment, or support and it is a serious threat to secondary prevention of HIV infection.

We found a study conducted among MSM in North Central Nigeria which investigated the impact of the law prohibiting such relationship in Nigeria [52]. The authors argued that there was increased fear of stigma and discrimination among this key population and as a result, access to care was affected.

### 3.4. Policies Related to HIV-Stigma in Nigeria

In Nigeria, policies and programmes for the national HIV response are coordinated by the National AIDS Control Agency (NACA). Other key partners include the Federal Ministry of Health (FMoH) as well as the State AIDS Control Agency (SACA) and Ministries of Health in the 36 states of the Federation. Policies are usually formulated at the national level and domesticated by the states. Though the administrative structure in Nigeria stipulates that states are federating units with autonomy to develop their own policies and programmes on issues such as health and education, there is a lot of synergy and cooperation among the federal and state government agencies. In this review, only policies and programmes enacted by the national government are reviewed because they apply across the entire country. Also, in order not to lose sight of the aim of this paper, we focused on the policy and programme components related to HIV-stigma.

#### 3.4.1. National HIV/AIDS Policy

The current HIV/AIDS policy in Nigeria was launched in 2009 [53]. Prior to that, similar policies have been formulated in 1997 and 2001. The 2009 policy document adopted a multisectoral approach to the fight against HIV in Nigeria. Therefore, its formulation involved extensive stakeholder engagement across public sector, private sector, and international development partners. Among the guiding principles of the 2009 policy was protection of rights of PLHIV and reduction of stigma and discrimination. The six strategic thrusts of the policy were (1) behaviour change and prevention of new infections; (2) treatment; (3) care and support for infected and affected persons; (4) institutional architecture and resourcing; (5) advocacy, legal issues, and rights; (6) monitoring and evaluation; (7) research and knowledge management. Stigma and discrimination were part of several policy objectives under strategic thrusts 1, 2, 3, and 5. The policy document clearly stated that “the rights of PLHIV include freedom from stigma and discrimination.” Stakeholders with stigma related responsibilities were national and state legislative assemblies (promoting policy dialogue and leading advocacy to reduce stigma and discrimination); faith-based organisations (advocacy for care and support; promoting stigma and discrimination reduction); traditional and religious leaders (supporting HIV/AIDS programmes and advocate for stigma reduction). Surprisingly, the policy document was silent on specific strategies to reduce stigma and discrimination under treatment, care, and support services.

#### 3.4.2. The 2010–2015 HIV/AIDS National Strategic Plan (NSP)

This was the third in a series of plans to combat HIV epidemic in Nigeria. The first plan captioned HIV/AIDS Emergency Action Plan (HEAP) 2001–2003 was developed in early 2001 and targeted at public awareness about the epidemic. Its successor was the National Strategic Framework for Action 2005–2009 developed in 2004/2005. It served as blueprint on implementation for different stakeholders. The 2010–2015 NSP [54] which has already completed its lifespan at the time of this review had six thematic areas which were lifted directly from the strategic thrust in the 2009 HIV/AIDS policy document. In the NSP, stigma and discrimination featured as an objective under care and support services. The strategic aim was “to reduce stigma and discrimination (S and D) against PLHIV and PABA by at least 60% of the baseline value by 2015.” Though the baseline year was 2010, the value is unknown. Thus, it is difficult to assess whether the target of 60% reduction has been met or not. Furthermore, S and D reduction was also listed among the activities to be carried out under policies, rights, and legal issues. Perhaps, the revision and eventual publication of the National HIV/AIDS Workplace policy in 2014 is an evidence of achievement on this note. Related to this are the passing and signing into law of the Nigeria's HIV/AIDS Antidiscrimination bill.

#### 3.4.3. National Workplace Policy on HIV and AIDS

The Nigeria National Workplace Policy on HIV and AIDS was first developed in 2005 to address the workplace response to the epidemic. The latest review was adopted and published in 2013 [55]. Such a policy became necessary because the population most affected by HIV in Nigeria are youths and those who are in the reproductive age groups. These also constitute the workforce of the country. Latest review was necessitated by three factors: changing epidemiology of HIV in the country; revision of the national HIV/AIDS policy which was its parent document; and implementation of the International Labour Organisation (ILO) recommendation 200 on HIV and AIDS in the workplace [56]. Nigeria is a signatory to several ILO recommendations and conventions. Guided by the 2009 National Policy on HIV and AIDS, the workplace policy document showed linkages with several other documents and legislations in Nigeria. One of the eight specific objectives of the policy focused on* “eliminating discrimination and stigmatization in the workplace based on real or perceived HIV status including dealing with HIV testing, confidentiality, and disclosure*.*”* The scope of the policy includes both public and private sectors including the uniformed services. Its guiding principles also conspicuously included* “nondiscrimination”* alongside nine others. To ensure that majority of Nigerians remain HIV-negative, the strategies itemised to achieve the objectives of the policy include prevention, HIV testing, treatment, care, and support, and promotion and protection of workers' rights. Responsibilities for the following stakeholders were also spelt out: Federal Ministry of Labour and Productivity, employers, workers, and their organisations, National Agency for Control of AIDS, State Agency for Control of AIDS, and National Steering Committee for HIV Workplace Issues. Curiously, implementation and action plans which were to be developed by different stakeholders are still being awaited.

#### 3.4.4. Nigeria's HIV and AIDS Antidiscrimination Act, 2014

First introduced to the National Legislature in 2005, the bill was passed and signed into law in April and November 2014, respectively. It serves a significant milestone in the national response to HIV/AIDS in Nigeria. This is because the nonpassage of the bill was a recurrent comment in the PLHIV stigma index and gender assessment report which are both reviewed in the next sections. The antidiscrimination act was very clear on rights of individuals [57]; their responsibilities; institutional obligations; and penalties for violations. It stipulates that PLHIV have rights to privacy about their serostatus, employment, welfare benefits, compensation, appeal, and recourse to courts as well as occupational safety. All Nigerians have responsibility to prevent stigma and discrimination. The responsibilities of PLHIV were disclosure of status to partner, demand for their right, and reporting of violations. Obligations of institutions, employers, and communities include protecting rights of PLHIV; providing equal opportunities to PLHIV; confidentiality of information; development of HIV/AIDS workplace policy; compensation for occupational HIV infection. Penalties for violations of the laws in the antidiscrimination act could be fines and/or imprisonment. Available information from the legal unit of NACA is that the act has been domesticated in 17 states across Nigeria. Two years after the law became active, there is a need for empirical evidence on the level of awareness; compliance; and the impact of the law on different aspects of the national HIV response in Nigeria. This is very crucial for the development of the next strategic response plan. Anecdotal evidence shows that lack of laws is not the main problem.

### 3.5. National HIV/AIDS Stigma Reduction Strategy, 2016

In response to the formidable threats posed by stigma and discrimination to the success of HIV prevention and treatment programmes, NACA in collaboration with other stakeholders developed the stigma reduction strategy for Nigeria [58]. Launched in November 2016, the goal of the strategy document was to “eliminate all forms of stigma and discrimination against people infected and affected by HIV in Nigeria by the year 2020.” It has nine strategic objectives with one contributing to the goal from different operational perspectives cutting across community-based organisations, faith-based organisations, media, entertainment and art industry, health and education institutions, and employers of labour (private and public), PLHIV and PABA. In other words, it was designed to achieve synergy among different players working with responsibilities to reduce/eliminate HIV-stigma with each entity leveraging on its comparative strengths in their jurisdictions, domains of coverage, and influence. Several strategic actions were listed including survey on behavioural, biomedical, and structural drivers of stigma and discrimination at different levels, increasing awareness about related laws. This national stigma reduction strategy actually integrated many of the UNAIDS suggestions on programmes and initiatives to promote stigma reduction at individual, community, structural, and institutional levels [2, 59].

### 3.6. Programmes Related to HIV-Stigma in Nigeria

Country annual report on the global response to HIV usually submitted to UNAIDS provides a comprehensive description of programmes implemented as part of the national response to HIV in Nigeria [4]. The commonest programme targeted at HIV-stigma and discrimination reduction in Nigeria is media and awareness campaigns aimed at educating the populace about HIV and ultimately reduce stigma and discrimination. Other approaches include the strategies deployed for universal counselling, testing, and treatment. The main philosophy of the approach was that if HIV is seen as a chronic condition just like hypertension or any other, then the infection would be better humanized thereby reducing stigma and discrimination against those infected and affected by HIV. Two other programmes related to HIV-stigma in Nigeria were the PLHIV stigma index measurement [60] and the Legal Environment Assessment in the HIV response [61].

#### 3.6.1. The PLHIV Stigma Index

The stigma index was designed to collect data on stigma, discrimination, and rights of people living with HIV. It was also aimed at serving as an advocacy tool and a way to operationalize the principle of Greater Involvement of People Living with HIV (GIPA). As of September 2013, it has been used in more than 50 countries including Nigeria. Further details on the development and processes involved in using the index are available on its dedicated website (www.stigmaindex.org).

In Nigeria, the index was applied by Networks of People Living with HIV and AIDS in Nigeria (NEPWHAN) in late 2009. Data was collected from 706 persons living with HIV selected from support groups in 12 states of the federation. The interviewers were also PLHIV. Report from the assessment documented several experiences of stigma and discrimination across three themes: exclusion, access to work, health, and educational services; internalised stigma; and fears [60]. Nearly one-third of respondents reported that they have been excluded from family and religious or other social functions due to their HIV status. A similar percentage also reported that they have experienced denial to health and educational services. Internalised stigma in form of shame, feeling of low self-esteem, and blaming oneself was reported by about 63%. In a nutshell, the index showed that stigma and discrimination experiences were prevalent among PLHIV in Nigeria. It is noted that the stigma assessment was done before the enactment of antidiscrimination law. Also, several programmes and initiatives have been implemented which must have influenced the narratives about HIV S and D in Nigeria. A repeat application of the stigma index is necessary to empirically assess the impacts of these various interventions.

### 3.7. Legal Environment Assessment for HIV/AIDS Response (LEA) in Nigeria

There has been a drive at international and regional levels to use laws as tools for HIV elimination. Particularly, to improve equity in access to services among key populations, law and regulatory environment must not be prohibitive. For instance, in Nigeria, HIV prevalence is higher among brothel-based female sex workers (19.4%), non-brothel-based female sex workers (8.6%), and men having sex with men (22.9%) [62]. Incidentally, there is a law prohibiting same-sex relationships in Nigeria. Empirical investigation of the effect of this law showed that it increased the incidence of stigma and discrimination against MSM such that they are afraid of accessing HIV care and treatment from public health facilities [52].

Stigma and discrimination are a main driver of inadequate access to care and treatment among these key populations. This would constitute a weak link in the HIV prevention efforts if an enabling environment is not created to protect this group against marginalisation and stigma.

The LEA in Nigeria was therefore designed to identify and review existing laws, regulations, and policies that could impact the national HIV response. Varieties of qualitative research methodologies (desk review, focus group discussion, in-depth interview, and key informant interview) were employed for the exercise. Findings showed that the legal environment in Nigeria is weak for effective human rights based response to HIV/AIDS [61]. Even though the 1999 constitution (amended) guaranteed rights of individuals against stigma and discrimination in any form, the institutional mechanisms for seeking redress could be easily exploited to the disadvantage of those in lower socioeconomic strata. Some of the legal provisions under different laws such as the criminal codes (which operates in Southern regions), penal codes (in the Muslim dominated northern regions), and same-sex marriage laws were found inconsistent [61]. While advocating for review of many of the existing laws related to stigma and discrimination, the LEA report also called for legal literacy among key populations and stakeholders in the justice sector.

## 4. Discussion

Although the measures used have not been consistent, there is evidence to show that the level of HIV-stigma in Nigeria has declined in the past two decades. This is demonstrated in results from analysis of attitudinal questions about people living with HIV in nationally representative surveys such as the NARHS 2003, 2005, 2007, and 2012 and NDHS 2013 [21, 23, 29, 63]. At least, early stories of isolation, abandonment, avoidance, and so forth have reduced drastically while uptake of HIV counselling and testing have improved [4]. HIV-stigma exists beyond the individual level and persists in families, communities, workplace, and other institutional contexts. Forms of HIV-stigma and discriminatory practices vary from one setting to another and this contextual difference is driven by economic and sociocultural diversity characteristic of Nigeria. At the base of this is the problem of power and inequality which visibly manifests as gender differences in HIV-stigma. Apart from the effect of stigma on testing, treatment uptake, adherence, care, and support, there is emerging evidence of a negative effect on treatment outcomes (quality of life and mental health). This review also found that PLHIVs have devised different positive and negative responses to stigma. Example of a positive response is by choosing to maintain good adherence so that physical health and overall quality of life can be like that of HIV-negative individuals. Another one is their family formation behaviour which is strongly motivated by a desire to demystify the misconceptions that fuel HIV-stigma and discrimination.

This review also revealed that the Government of Nigeria with support from development partners have done a lot to confront HIV-stigma. National policies and strategic plans have been revised about three times to align with international best practices as well as respond to the epidemiological and structural dynamics of the HIV epidemic in the country. A robust multisectoral approach with stakeholders involvement, political will, and policy advocacy at national and state levels have all contributed to general awareness about the urgent need to eliminate stigma and curtail HIV spread. To further improve on the successes recorded so far, some recommendations are made which could strengthen the stigma reduction programmes in the country.

There is a need for reliable, validated empirical measures of HIV-stigma. Even though the Berger HIV-stigma scale was applied at a facility in SW Nigeria [46], its internal consistency (reliability) was just average. Measures already validated in other SSA settings [12] can be revalidated in the country. Availability of reliable and validated measures will facilitate objective assessment which is very important for the monitoring of the recent stigma reduction strategy in the country. The need to monitor the impact of the 2014 antidiscrimination law also makes this necessary.

Another reason for new measures of stigma stems from the scale-up of ART services to secondary and primary healthcare facilities. It is expected that a deeper population penetration of ART would help correct the wrong belief that HIV is a death sentence. This misconception has been responsible for abandonment in the early 2000s. It also heightened the fear of casual transmission via mere physical contact. Now that treatment is closer to the grassroots, it ought to have indirectly mitigated some of the stigma. Operational research studies need to be undertaken to explore this proposition.

Much of what is known about HIV-stigma in Nigeria have come largely from research studies in North Central, South East, South West, and South South regions. Considering the evidence about the dynamic sociocultural nature of stigma, there is need for complimentary evidence from the North East and North West regions. The case for the North East is even more sacrosanct because of the internal population displacement occasioned by the Boko Haram insurgency. There is anecdotal evidence of increased new HIV infections in internally displaced persons (IDP) camps. Although some of these internally displaced persons are being resettled in their communities, the level of HIV-stigma in IDP camps must have been enormous. The experiences of those affected and how they coped with the challenges would provide useful lessons for stigma reduction programmes in Nigeria.

Another implication of the preponderance of HIV-stigma studies in Nigeria being domiciled in tertiary health facilities is that they also provide information about urban areas. Could stigma be higher in rural areas? Evidence from population-based surveys suggests that stigmatizing attitudes towards PLHIV was associated with lower education, poverty, and poor knowledge of HIV [23, 63]. These features are characteristic of rural settings in Nigeria. In addition, the only facility-based study among health workers in primary health centres also showed that stigma was high among this cadre of health workforce [38]. These are kinds of facilities that exist in most rural areas. Therefore, it can be expected that stigma will be higher in rural areas.

The legislative environment in Nigeria is not favourable to key populations at risk of HIV such as MSM. This may also explain why investigation of HIV-stigma in this population is rare. The only study among this group showed that enactment of the law prohibiting same-sex partnership in Nigeria has contributed to higher level of fear [52], although, prior to the legislation, the existing social environment cannot be claimed to be conducive to MSM. Therefore, it is difficult to draw a definitive conclusion about the effect of the new law.

## 5. Conclusion

Without prejudice to other strategies aimed at eliminating HIV by year 2030, stigma and discrimination reduction is a priority. Research, policies, and programmes in the past decade have made tremendous contributions to this drive. Newer approaches tailored to the epidemiological and social contexts of individual countries would continue to evolve. In this regard, this paper reviewed research studies, policies, and programmes related to HIV stigma in Nigeria, the country with the second largest number of people living with HIV in the world. This review identified the need for a consistent valid and objective measure of stigma at different levels of the HIV response. Empirical evidence on the awareness and effect of anti-HIV discrimination law and other interventions are urgently needed. Nigeria is not lacking in policies as this review shows that the country is nearly up to date in compliance with UNAIDS guidelines on several policy requirements. What need to be strengthened are programmes design, planning, monitoring, and evaluation. It is necessary to intensify advocacy, awareness, and enforcement of the anti-HIV discrimination law. It is also very important to develop systems for evaluating the impact of stigma and discrimination reduction programmes at national and subnational levels.

## Figures and Tables

**Table 1:** Literature matrix of studies on HIV-stigma in Nigeria: 2002–2016.

SN	Ref: author (year)	Study design	Study population and location	Objectives
(1)	1999: Fawole et al.	Quasi-experimental study	Secondary students in South West Nigeria	To assess the effect of educational intervention on attitudes of students to people living with HIV/AIDS

(2)	2000: Uwakwe	Quasi-experimental study	Student nurses in SW Nigeria	To investigate the effect of educational program on attitudes and behaviour regarding HIV/AIDS

(3)	2002: Alubo et al.	Qualitative study	Community members in southern part of Benue State, North Central Nigeria	To document the perceptions of PLHIV, family, and community members about HIV stigma

(4)	2002: Ezedinachi et al.	Quasi-experimental study	Facility-based among health workers in South South Nigeria	To assess the effect of educational intervention on knowledge and attitudes of workers to PLHIV

(5)	2003: Adebajo et al.	Cross-sectional survey	Nurses and medical lab scientists in public health facilities in Lagos, South West, Nigeria	To document attitudes to PLHIV

(6)	2005: Adedigba et al.	Cross-sectional study	Oral health workers in Osun State, South West Nigeria	Assessed KAP of infection control in managing HIV-infected patients

(7)	2005: Reis et al.	Cross-sectional survey	Health workers at a tertiary facility in 4 states of Nigeria	To assess the extent of discrimination against PLHIV

(8)	2006: Adeokun et al.	HIV surveillance project	Market women in Ibadan and Ogbomosho, South West Nigeria	To assess the effect of behavioural change intervention on HIV stigma and discrimination

(9)	2007: Babalola	Cross-sectional survey	Population-based sample, Bauchi (North East) and Kano (North West), Nigeria	To assess the relationship between individual perceived stigma, community stigma (social norm), and attitude to VCT. Male and females were analysed separately Stigma was used as main IV

(10)	2008: Bukar et al.	Cross-sectional study among	Dental professionals in one state in each region of Nigeria	To investigate discrimination against PLHIV among dental professionals

(11)	2008: Lapinski and Nwulu	Field experiment: Group 1 exposed to a film versus another group not exposed	Community-based study, Abuja, North Central Nigeria	The assess the efficacy of an education-entertainment intervention (film) on HIV risk perceptions and perceived stigma

(12)	2009: Adewuya et al.	Cross-sectional study	PLHIV at a private HIV/AIDS care centre in South West Nigeria	Determine prevalence and factors associated with PTSD induced by a stigmatising event among PLHIV

(13)	2009: Babalola et al.	Secondary analysis of cross-sectional survey-National HIV/AIDS and Reproductive Health Survey (NARHS) 2005	Population-based nationally representative sample	To determine the relationship between media exposure, community media saturation, and accepting attitudes to PLHIV in Nigeria

(14)	2009: Sadoh et al.	Cross-sectional study	Healthcare workers at a facility in South Eastern Nigeria	To document the attitude of health workers to colleagues infected with HIV

(15)	2010: Fakolade et al.	Secondary analysis of NARHS 2003, 2005, and 2007 data	Population-based nationally representative sample	Assess the impact of mass media exposure on HIV stigma and discrimination

(16)	2010: Mbonu et al.	Qualitative method: in-depth interview (IDI)	Community members, healthcare workers and PLHIV in Port-Harcourt, South South Nigeria	To explore power-related differences between men and women in their reactions to people living with HIV/AIDS at family, community, and societal (institutional) level

(17)	2010: Smith and Mbakwem	IDI and observation in 2004, 2006, and 2007	PLHIV selected from a tertiary treatment facility in Owerri, South East Nigeria	To explore the reproductive life of HIV-positive men and women and understand how they balance medical advice with societal expectations about marriage and reproduction

(18)	2011: Adebayo et al.	Secondary analysis of NARHS 2003, 2005, and 2007 data	Population-based nationally representative sample	Explore levels, trends, and geographical variations in accepting attitude to PLHIV (stigma measure)

(19)	2011: Winskell et al.	Textual analysis of narratives from a writing contest on HIV stigma	Youth participants, Enugu, South East Nigeria	Compare social representation of symbolic stigma (moralistic blaming and shaming of those infected) across 6 countries

(20)	2012: Owolabi et al.	Cross-sectional study	PLHIV at a tertiary treatment facility in Ilorin North Central, Nigeria	Describe the level of stigma and discrimination faced by ART patients

(21)	2013: Blessed and Ogbalu	Cross-sectional study	PLHIV at a tertiary facility in SE Nigeria	Describe the forms of stigma experienced by PLHIV

(22)	2013: Kavanaugh et al.	Cross-sectional study	Community-based sample; Lagos (South West), Benue (North Central), Kaduna (North West), and Enugu (South West)	Describe community attitudes towards possible outcomes of pregnancy by HIV-positive women (abortion or birth)

(23)	2013: Odimegwu et al.	Mixed-method cross-sectional study	Community-based; Osogbo (South West) and Orlu (South East) Nigeria	Describe attitude of community members to PLHIV and how this affects VCT uptake

(24)	2013: Okoror et al.	Qualitative exploration (FGD and IDI)	Community-based organisation in South West Nigeria	Explore how stigma affects ART adherence and the effect of ART on perceived stigma

(25)	2013: Sekoni and Owoaje	Cross-sectional study	Healthcare workers at Primary Healthcare Centres in Kwara State, North Central Nigeria	To investigate stigma and discrimination against PLWHA among PHC workers

(26)	2014: Aransiola et al.	Qualitative study	PLHIV at a secondary health facility in South West Nigeria	To explore stigma and other survival challenges among ART patients

(27)	2014: Olalekan et al.	Qualitative study	PLHIV at a facility in Lagos, South West Nigeria	To explore the perceptions of PLHIV about societal stigma associated with HIV

(28)	2014: Omosanya et al.	Cross-sectional study	PLHIV, tertiary facility in South West Nigeria	To investigate the relationship between HIV stigma and ART adherence

(29)	2014: Onyebuchi-Iwudibia and Brown	Cross-sectional study	PLHIV; treatment facility in Enugu, South East Nigeria	To determine prevalence of depression and its association with HIV-related stigma

(30)	2015: Dahlui et al.	Secondary analysis of Nigeria Demographic and Health Survey 2013 data	Population-based nationally representative sample	Describe the level of stigma against PLHIV in Nigeria

(31)	2015: Schwartz et al.	Longitudinal study among	MSM at a secured clinic setting in Abuja, North Central Nigeria	To investigate the effect of the same-sex prohibition act on stigma and discrimination against MSM in Nigeria

(32)	2016: Olley et al.	Cross-sectional study	PLHIV, tertiary treatment facility in Akure, South West, Nigeria	To investigate the mediating or moderating role of anticipated discrimination in the relationship between HIV-related stigma and status disclosure
